# Neoadjuvant chemotherapy (NCT) plus targeted agents versus NCT alone in colorectal liver metastases patients: A systematic review and meta-analysis

**DOI:** 10.18632/oncotarget.5875

**Published:** 2015-10-22

**Authors:** Chun-Hui Cui, Shu-Xin Huang, Jia Qi, Hui-Juan Zhu, Zong-Hai Huang, Jin-Long Yu

**Affiliations:** ^1^ Department of General Surgery, Zhujiang Hospital, Southern Medical University, Guangzhou, Guangdong Province, China; ^2^ Department of Ophthalmology, Zhujiang Hospital, Southern Medical University, Guangzhou, Guangdong Province, China

**Keywords:** neoadjuvant chemotherapy, targeted agents, colorectal liver metastasis, meta-analysis

## Abstract

**Purpose:**

To assess the efficacy of neoadjuvant chemotherapy (NCT) plus targeted agents versus NCT alone for the treatment of colorectal liver metastases (CRLM) patients.

**Methods:**

Trials published between 1994 and 2015 were identified by an electronic search of public databases (MEDLINE, EMBASE, Cochrane library). All clinical studies were independently identified by two authors for inclusion. Demographic data, treatment regimens, objective response rate (ORR), hepatic resection and R0 hepatic resection rate were extracted and analyzed using Comprehensive MetaAnalysis software (Version 2.0).

**Results:**

A total of 40 cohorts with 2099 CRLM patients were included: 962 patients were treated with NCT alone, 602 with NCT plus anti-epidermal growth-factor receptor (EGFR)-monoclonal antibodies (MoAbs) and 535 with NCT plus bevacizumab. Pooled ORR was significantly higher for NCT plus bevacizumab or anti-EGFR-MoAbs than NCT alone [relative risk (RR) 1.53, 95% CI 1.30–1.80; *p* < 0.001; RR 1.53, 95% CI: 1.27–1.83, *p* < 0.001; respectively]. NCT plus bevacizumab significantly improved R0 hepatic resection rate (RR 1.61, 95% CI: 1.27–2.04, *p* < 0.001), but not for overall hepatic resection rate (RR 1.26, 95% CI: 0.81–1.94, *p* = 0.30). While hepatic resection and R0 hepatic resection rate was comparable between NCT plus anti-EGFR-MoAbs and NCT alone (*p* = 0.42 and *p* = 0.37, respectively).

**Conclusions:**

In comparison with NCT alone, NCT plus bevacizumab significantly improve ORR and R0 hepatic resection rate but not for hepatic resection rate. Our findings support the need to compare NCT plus bevacizumab with NCT alone in the neoadjuvant setting in large prospective trials due to its higher hepatic resection rate and R0 hepatic resection rate in CRLM patients.

## INTRODUCTION

Colorectal cancer (CRC) is one of the most common malignant tumors throughout the world with over 1.2 million new cases and 608700 deaths estimated to occur annually [1]. The liver is the most common site of colorectal cancer metastasis. Nearly half of CRC patients will develop colorectal liver metastases (CRLM) during the course of their disease, with 15% of patients having liver metastatic lesions at the time of diagnosis [2]. Surgical resection of colorectal liver metastases is a potentially curative option, with reported 5-year survival of 28–39% [3–5] and 10-year overall survival of over 20% [4, 6]. However, unfortunately, 70–80% of patients will relapse in two years after liver surgery, and about 80% of patients with colorectal liver metastases have unresectable disease at presentation [7].

To improve the prognosis of CRLM patients, it is important to improve the liver metastasis treatment outcomes. Over the past decade, the introduction of irinotecan- or oxaliplatin-based combination chemotherapy have resulted in significant improvements in objective response rates and ultimately in overall survival of unselected patients with metastatic colorectal cancer [8–10]. In recent years, neoadjuvant chemotherapy (NCT) has been increasingly used in the management of liver-confined metastases from CRC. For patients with initially resectable disease, the use of NCT in CRLM might increase the complete resection rate and treat the micro-metastatic disease [3, 11]. When treating unresectable liver metastases of colorectal cancer, “conversion therapy” has been applied to reduce the tumor size and facilitate resection via preoperative chemotherapy [12, 13]. In addition, NCT can be used as a test of *in vivo* chemosensitivity, and patients with extremely aggressive disease, who will progress during preoperative chemotherapy, can be spared useless surgery. As a result, neoadjuvant chemotherapy combined surgery for liver metastasis is regarded as an effective strategy in CRLM patients.

During the past decade, the understanding of the molecular pathways that involved in tumor growth and metastasis has significantly increased and with this has come the development of several molecular targeted therapies [9, 14–16]. Two options are currently available in routine clinical practice for CRLM patients: Epidermal growth factor receptors (EGFRs) antibodies and vascular endothelial growth factor (VEGF) antibodies. The efficacy of these molecular targeted agents in the treatment of unselected metastatic CRC has been extensively investigated, but whether the addition of molecular targeted agents to NCT in CRLM patients would improve response rate and hepatic resection rate remains unclear. A recent meta-analysis conducted by Qi et al [17] showed that the addition of targeted agents to first-line chemotherapy for unselected advanced colorectal cancer significantly improved the complete response when compared with controls. However, it is still unknown whether this benefit in response rate would translate into an improvement in hepatic resection rate and R0 hepatic resection rate for CRML patients. We thus conduct this meta-analysis of published data to compare the efficacy of NCT plus targeted agents verse NCT alone in CRLM patients.

## RESULTS

### Search results

**Figure 1:**
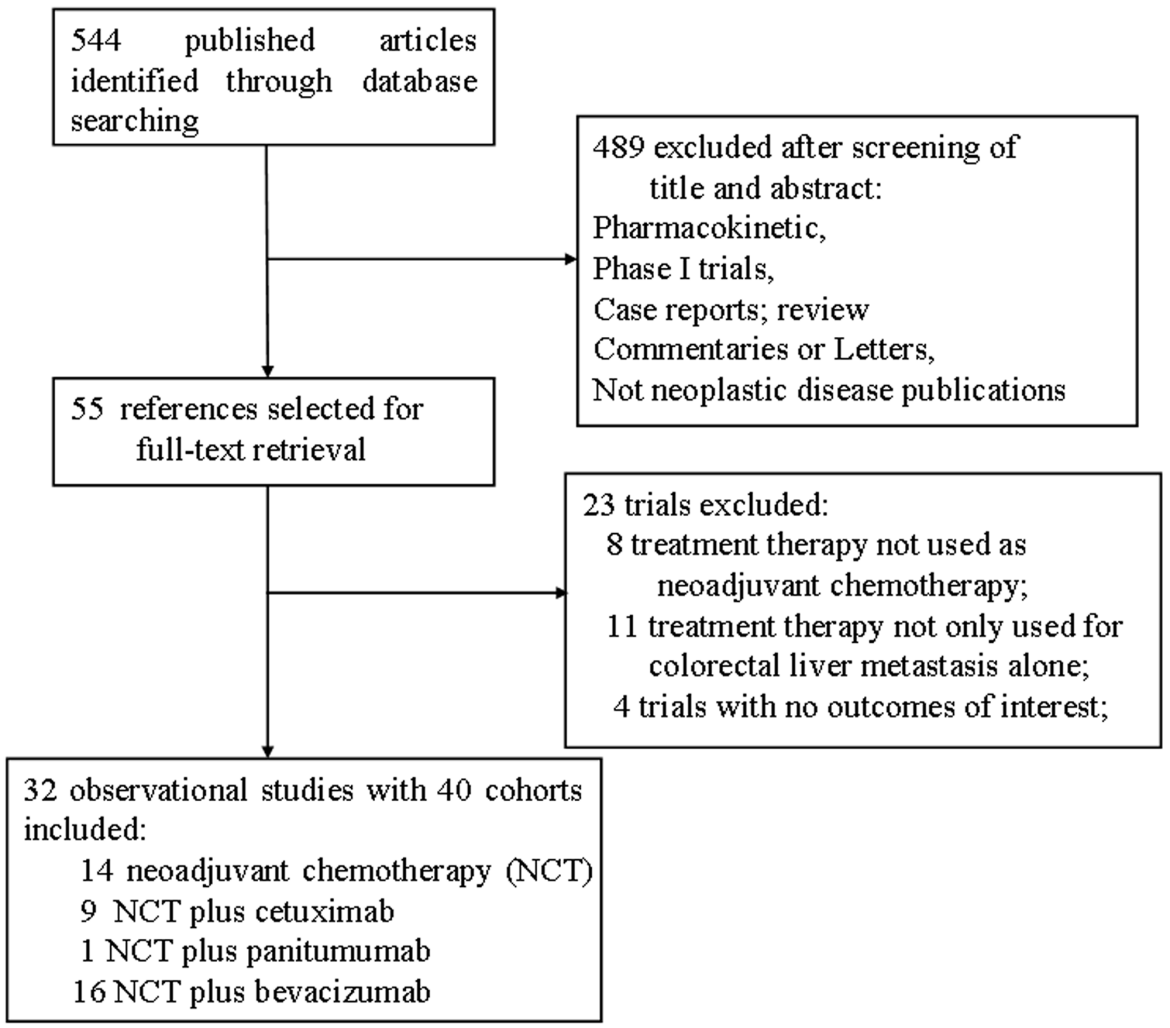
Selection process for clinical trials included in the meta-analysis

**Figure 2:**
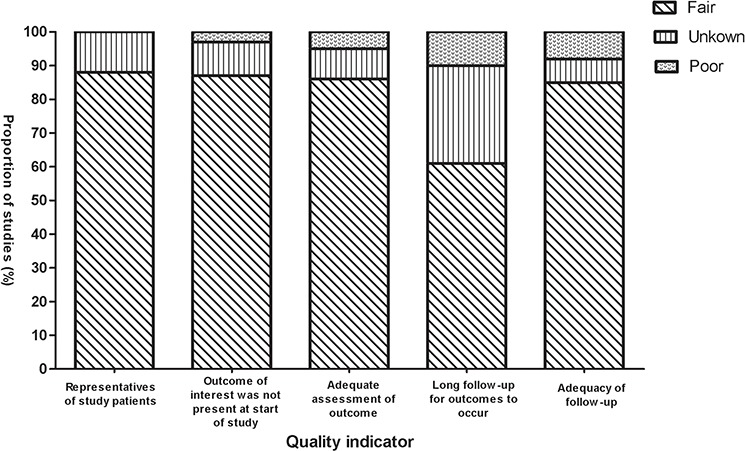
Selected methodological quality indicator

A total of 543 studies were identified from the database search, of which 54 reports were retrieved for full-text evaluation. 40 cohorts from 32 trials [24–54] met the inclusion criteria and were included in this systematic review: (Figure 1). Table 1 showed the characteristics of the included studies. Overall, 2099 CRLM patients were included, with a median age of 62.0 years [95% confidence interval (CI): 59.0–62.91] for the NCT alone group and 61.0 years (95% CI: 58.2–62.9) for the NCT plus targeted agents group. We found two randomized controlled trials comparing NCT plus cetuximab with NCT alone in CRLM patients, but no randomized controlled trials directly comparing NCT plus bevacizumab with NCT alone in these settings. Methodological quality of the included studies was fair; most studies provided adequate outcome ascertainment, enrolled a representative sample of patients, and had an acceptable length of follow-up (Figure 2). However, comparative evidence was at high risk of bias because we compared data across studies not within them, and selection bias was likely to be present. Assessment of publication bias was not done because data would be unreliable in view of the few studies included for each treatment group and high heterogeneity (*I*2 > 50%) in most analyses.

**Table 1:** Baseline characteristics of 39 cohort groups for meta-analysis

Author	Year	Study design	Patients, n	Neoadjuvant therapy	Median age, y	Initially status, n	Median PFS, months	ORR, %
Uetake H. et al	2015	Prospective	45	mFOLFOX6+bevacizumab	62.5	Resectable,19 unresectable, 26	NR	55.60%
Suenaga M. et al	2015	Prospective	12	FOLFOX4+bevacizumab	60.5	Unresectable	18.2	75%
Pietrantonio F. et al	2015	Retrospective	93	Chemotherapy +bevacizumab	56	NR	NR	78%
				Chemotherapy +cetuximab	59	NR	NR	85%
Malik H. et al	2015	Retrospective	60	Chemotherapy +cetuximab	62	Unresectable	NR	NR
Gruenberger T. et al	2015	Prospective	80	FOLFOXIRI +bevacizumab	63	Unresectable	18.6	81%
				mFOLFOX6+bevacizumab	57	Unresectable	11.5	62%
Vera R et al	2014	Retrospective	95	Chemotherapy +bevacizumab	NR	Unresectable	NR	52%
				chemotherapy	NR	Unresectable	NR	50%
Primrose J. et al	2014	Prospective	257	Chemotherapy +cetuximab	63	Resectable	14.1	70%
				chemotherapy	64	Resectable	20.5	62%
Eppu T. et al	2014	Prospective	40	FOLFOX6+bevacizumab	63	NR	9.7	30%
Ychou M. et al	2014	Prospective	125	chemotherapy	NR	Unresectable	11.9	NR
Takahashi T. et al	2013	Prospective	36	mFOLFOX6	62.5	Unresectable	9.2	NR
Nordlinger B et al.	2013	Prospective	171	FOXFOX4	62	Resectable	NR	NR
Ye L.C. et al	2013	Prospective	138	Cetuximab +chemotherapy	57	Unresectable	NR	57.10%
				chemotherapy	59	Unresectable	NR	29.40%
Nasti G. et al	2013	Prospective	39	FOLFIRI +Bevacizumab	58	Resectable	14	66.70%
Ji J.H. et al	2013	Prospective	73	FOLFOX6+cetuximab	57	Unresectable	9.8	72.60%
Cvetanovic A. et al	2013	Retrospective	51	Oxaliplation-based +bevacizumab	NR	NR	9.9	NR
Constantinidou A. et al	2013	Retrospective	94	Chemotherapy +bevacizumab	63	NR	NR	NR
				chemotherapy	62	NR	NR	NR
Leone F. et al	2013	Prospective	46	Panitumumab +XELOX	60	Unresectable	8.5	54%
Wong R. et al	2011	Prospective	46	Xelox +bevacizumab	63	Unresectable, 30	NR	78%
Bertolini F. et al	2011	Prospective	21	FOLFOX6+bevacizumab	NR	Unresectable	12.5	57%
Nakanishi M. et al	2014	Retrospective	20	Bevacizumab +chemotherapy	NR	Resectable	NR	66.70%
Garufi C. et al	2010	Prospective	43	Chemotherapy +cetuximab	61	Unresectable	NR	79%
Folprecht G. et al	2010	Prospective	111	FOLFOX6+cetuximab	65.1	NR	NR	68%
				FOLFIRI+ cetuximab	62	NR	NR	57%
Chaudhury P. et al	2010	Retrospective	35	Chemotherapy +bevacizumab	57	NR	NR	65.70%
Masi G. et al	2010	Prospective	30	FOLFOXIRI +bevacizumab	61	Unresectable	16.9	80%
Skof E. et al	2009	Prospective	87	XEFIRI	63	Unresectable	10.3	NR
				FOLFIRI	62	Unresectable	16.6	NR
Bathe O. et al	2009	Prospective	35	FOLFIRI	59	Resectable	NR	NR
Coskun U. et al	2008	Retrospective	35	XELOX	58	Unresectable	NR	NR
Barone C. et al	2007	Prospective	40	FOLFIRI	58.7	Unresectable	14.3	NR
Gruenberger B. et al	2008	Prospective	56	Xelox +bevacizumab	61.5	Resectable	NR	73%
Min B.S. et al	2007	Prospective	23	FOLFIRI +cetuximab	NR	Unresectable	NR	39.10%
Alberts S.R. et al	2005	Prospective	42	FOLFOX	63	Unresectable	NR	NR
Wein A. et al	2003	prospective	20	FOLFOX	62.5	Resectable	NR	NR

Abbreviations: PFS, progression free survival; ORR, objective response rate; FOXFOX, oxaliplatin plus leucovorin plus fluorouraci; FOLFIRI, irinotecan plus leucovorin plus fluorouraci; Xelox, xeloda plus oxaliplatin; XEFIRI, xeloda plus irinotecan; FOLFOXIRI, irinotecan plus oxaliplatin plus leucovorin plus fluorouraci; NR, not reported;

### Pooled incidence of primary outcomes

**Figure 3:**
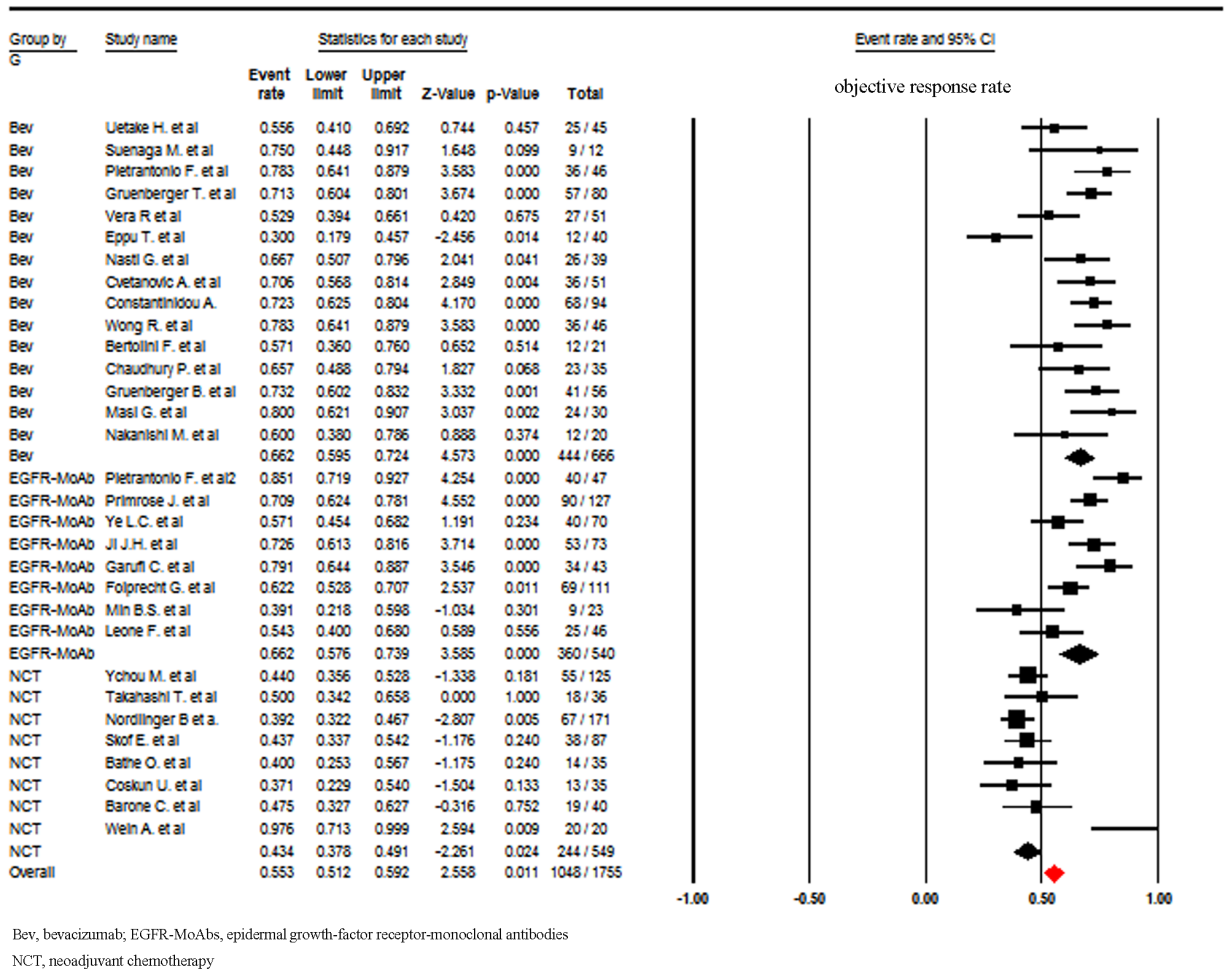
Incidence of objective response rate according to neoadjuvant regimens

**Figure 4:**
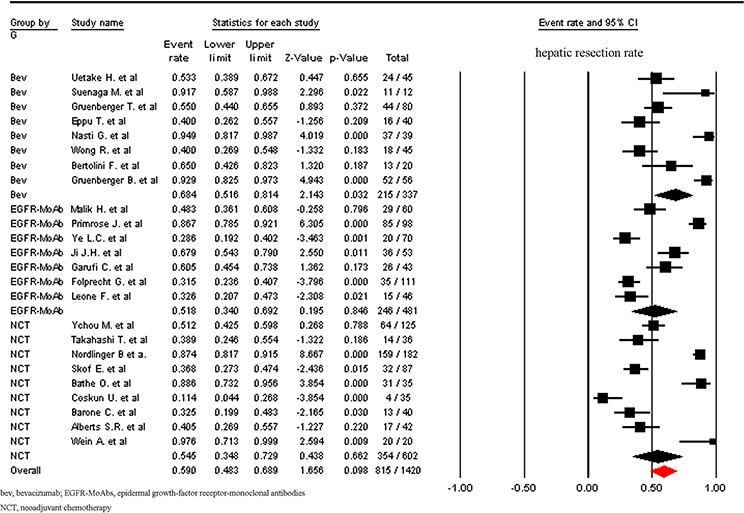
Incidence of hepatic resection rate according to neoadjuvant regimens

**Figure 5:**
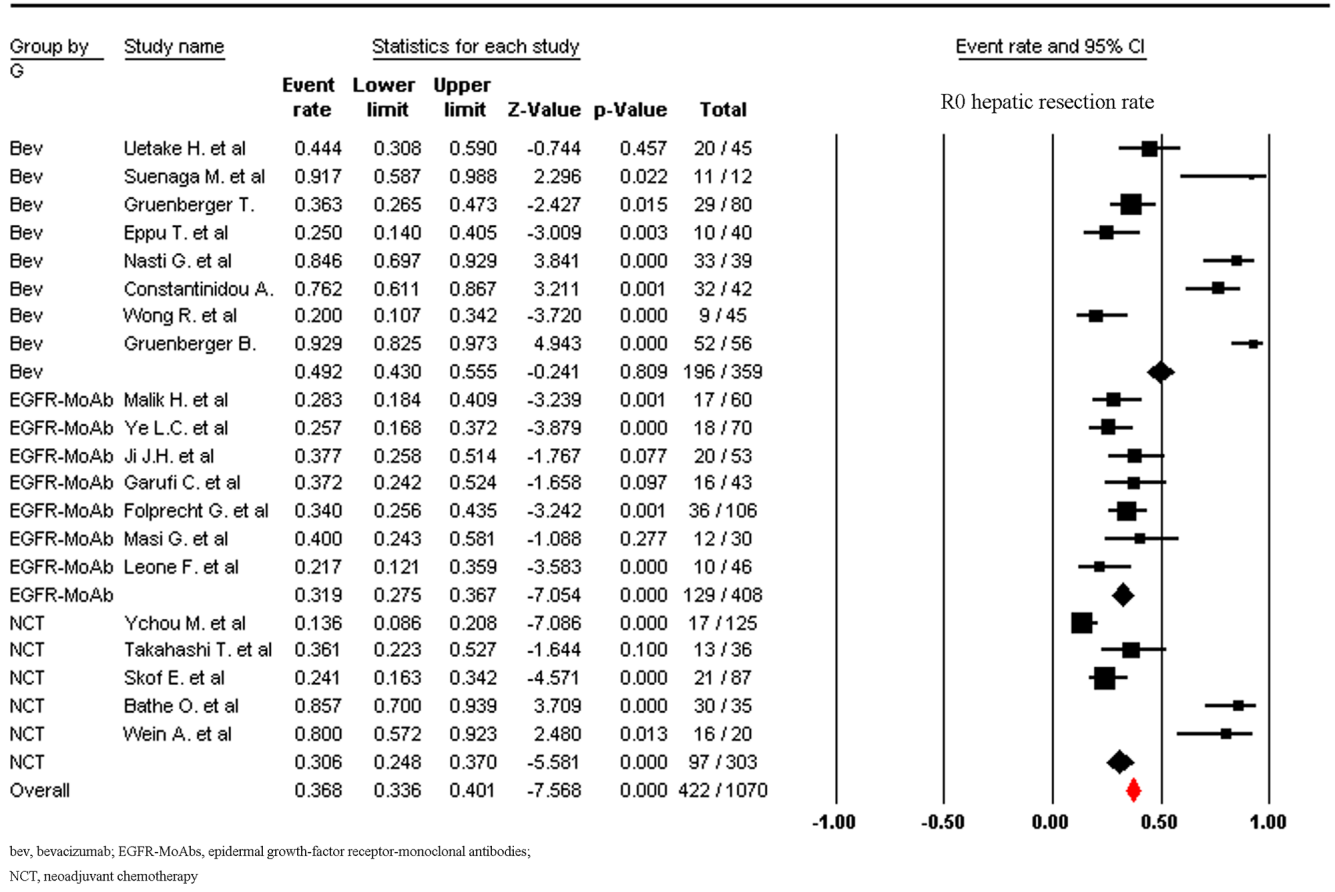
Incidence of R0 hepatic resection rate according to neoadjuvant regimens

A total of 1755 patients were included for ORR analysis. The pooled event rate of ORR for NCT plus bevacizumab and NCT plus anti-epidermal growth-factor receptor (EGFR)-monoclonal antibodies (MoAbs) were 66.2% and 66.2% respectively, which was higher than that of NCT alone (43.4%, Figure 3). A higher incidence of hepatic resection and R0 hepatic resection was observed in NCT plus bevacizumab (68.4% and 49.2% respectively) when compared to NCT plus anti-EGFR-MoAbs or NCT alone. While comparable incidence of hepatic resection and R0 hepatic resection was found between NCT plus anti-EGFR-MoAbs and NCT alone (Figures 4 and 5).

### Efficacy comparison between NCT plus targeted agents and NCT

In comparison with NCT alone, NCT plus bevacizumab or anti-EGFR-MoAbs significantly improve ORR (RR 1.53, 95% CI 1.30–1.80; *p* < 0.001; RR 1.53, 95% CI: 1.27–1.83, *p* < 0.001; respectively), NCT plus bevacizumab significantly improved R0 hepatic resection rate in comparison with NCT alone (RR 1.61, 95% CI: 1.27–2.04, *p* < 0.001), but not for overall hepatic resection rate (RR 1.26, 95% CI: 0.81–1.94, *p* = 0.30). While hepatic resection rate and R0 hepatic resection rate was comparable between NCT plus anti-EGFR-MoAbs and NCT alone (* p* = 0.42 and *p* = 0.37, respectively) (Table 2).

**Table 2:** Comparison of primary outcomes for NCT plus target agents versus NCT alone

Groups	Cohorts (n)	Patients (n)	Events (95%)	*I*^2^	Relative risk (95%)	*p*
ORR						
NCT	8	549	43.4 (37.8–49.1)	33.5	1	−
NCT plus bevacizumab	15	666	66.2 (59.5–72.4)	64.5	1.53 (1.30–1.80)	<0.001
NCT plus EGFR- MoAb	8	560	66.2 (57.6–73.9)	73.3	1.53 (1.27–1.83)	<0.001
Hepatic resection rate						
NCT	9	602	54.5 (34.8–72.9)	93.5	1	−
NCT plus bevacizumab	8	337	68.4 (51.6–81.4)	85.2	1.26 (0.81–1.94)	0.30
NCT plus EGFR- MoAb	7	481	51.8 (34.0–69.2)	92.5	0.95 (0.57–1.59)	0.42
R0 hepatic resection rate						
NCT	5	216	30.6 (24.8–37.0)	93.6	1	−
NCT plus bevacizumab	8	285	49.2 (43.0–55.5)	91.7	1.61 (1.27–2.04)	<0.001
NCT plus EGFR- MoAb	7	232	31.9 (27.5–36.7)	3.2	1.04 (0.81–1.33)	0.37

*I*^2^ ≥ 50% suggests high heterogeneity across studies.

Abbreviation: NCT = neoadjuvant chemotherapy; ORR, objective response rate;

### Sub-group analysis

Six included trials reported efficacy data about anti-EGFR-MoAbs according to K-ras status in CRLM patients. The pooled ORR, hepatic resection and R0 hepatic resection rate for CRLM with K-ras wild-type receiving EGFR-MoAbs were 64.6% (95% CI: 59.8–69.0%), 56.7% (95% CI: 33.7–77.2%), and 30.0% (95% CI: 24.2–36.4%), respectively. Then, we performed sub-groups analysis according to respectability status. For initially resectable CRLM patients, the addition of targeted agents to NCT did not significantly improve hepatic resection rate (88.9% versus 82.5%) and R0 hepatic resection rate (67.5% versus 69.7%), while the addition of targeted agents to NCT increased ORR (65.0% versus 44.3%), hepatic resection rate (54.8% versus 35.5%) and R0 hepatic resection rate (38.0% versus 18.3%) in comparison to NCT alone (Table 3). Additionally, we performed sub-groups analysis based on combined chemotherapy. Irinotecan-based NCT plus targeted agents seemed to improve hepatic resection rate and R0 hepatic resection rate when compared to NCT alone (Table 3). However, only one trial investigating FOLFIRI plus bevacizumab in resectable CRLM patients was included for analysis, thus further studies were still needed to assess the efficacy of irinotecan-based NCT plus targeted agents in CRLM patients. For CRLM patients receiving oxaliplatin-based NCT, the addition of targeted agents to oxaliplatin-based chemotherapy increased ORR (65.1% versus 46.3%), but not for hepatic resection rate and R0 hepatic resection rate (Table 3).

**Table 3:** Sub-group analysis of efficacy for NCT plus target agents versus NCT alone

Groups	ORR		Hepatic resection rate		R0 hepatic resection rate	
Initial status	NCT	NCT plus targeted agents	NCT	NCT plus targeted agents	NCT	NCT plus targeted agents
Resectable	48.8% (27.3–70.7%)	68.2% (61.5–74.2%)	82.5% (50.2–95.6%)	88.9% (82.7–93.1%)	69.7% (31.7–92.0%)	67.5% (21.5–94.0%)
Unresectable	44.3% (39.0–49.8%)	65.0% (62.3%-71.0%)	35.5% (24.7–48.0%)	54.8% (42.1–66.9%)	18.3% (10.1–30.9%)	38.0% (26.7–50.8%)
Chemotherapy						
Irinotecan-based	43.8% (36.4–51.6%)	55.4% (41.4–68.7%)	54.3% (24.5–81.4%)	94.9% (81.7–98.7%)	57.3% (7–96%)	84.6% (69.7–92.9%)
Oxaliplatin-based	46.3% (31.4–61.8%)	65.1% (58.3–71.3%)	57.8% (22.3–86.7%)	53.3% (48.8–57.8%)	58.9% (17.4–90.7%)	36.5% (31.9–41.3%)

Abbreviation: NCT, neoadjuvant chemotherapy; ORR, objective response rate;

## DISCUSSION

The liver is the common metastatic site for colorectal cancer, and surgical resection is the only therapeutic modality that offers the potential for long-term cure. Appropriate patient selection for surgery and improvements in perioperative care has resulted in low morbidity and mortality rates, meaning that this is the therapy of choice in suitable patients. In recent years, the survival of patients with metastatic colorectal cancer has been improved, initially by the use of oxaliplatin- or irinotecan-based combination chemotherapy. Subsequently, it has been shown that the efficacy of cytotoxic chemotherapy can be enhanced by the addition of novel targeted agents, notably the anti-VEGF monoclonal antibodies and the anti-EGFR antibodies. However, the efficacy of targeted agents in neoadjuvant chemotherapy for CRLM patients remains unknown. Recently, two prospective randomized controlled trials have been conducted to investigate the efficacy of NCT plus cetuximab versus NCT alone in CRLM patients [33]. The trial conducted by Ye L.C et al showed that cetuximab combined with chemotherapy improved the resectability of liver metastases and improved response rates (57.1% versus 29.4%) and 3-year survival (41% versus 18%) in comparison with chemotherapy alone for K-RAS wild-type CRLM patients [33], while the New EPOC trial showed that the addition of cetuximab to neoadjuvant chemotherapy for K-RAS wild-type CRLM patients resulted in shorter progression-free survival (HR 1.48, 95% CI: 1.04–2.12, *p* = 0.03) [29]. Therefore, the role of cetuximab in neoadjuvant setting for CRLM patients is not established. In addition, to the best of our knowledge, there is lack of head-to-head comparison data available for NCT plus bevacizumab versus NCT alone in the treatment of CRLM patients. As a result, we conduct this systematic review and meta-analysis to evaluate the efficacy of NCT plus targeted agents versus NCT alone for the treatment of CRLM patients.

A total of 2099 CRLM patients from 40 cohorts are included for analysis. Based on our pooled results, we find that NCT plus bevacizumab could employ a role in the neoadjuvant setting for CRLM patients in particular in terms of ORR and R0 hepatic resection rate, while comparable efficacy is found between NCT plus EGFR-MoAbs and NCT alone in terms of hepatic resection rate and R0 hepatic resection rate. Additionally, several retrospective studies have demonstrated that the addition of bevacizumab to chemotherapy in the neoadjuvant and conversion setting significantly improve pathological response in CRLM patients, and patients with a good pathological response to neoadjuvant chemotherapy are associated with a better outcome [41, 55, 56]. Based on these encouraging data, the combination of NCT plus bevacizumab as neoadjuvant and conversion therapy could be recommended for initially unresectable or resectable CRLM patients due to its higher ORR and R0 hepatic resection rate. However, more evidence is still required before NCT plus bevacizumab could become the standard peri-operative treatment for these patients.

We then perform sub-group analysis according to patients' characteristics. Our results find that the addition of targeted agents to NCT seems more efficient for initially unresectable patients than for initial resectable patients in terms of hepatic resection rate and R0 hepatic resection rate. It might be explained that the addition of targeted agents to NCT could increase the efficacy of neoadjuvant treatment in initially unresectable CRLM patients, which might achieve maximum tumor shrinkage to create an opportunity for hepatic resection. and it has been reported that rate of early tumor shrinkage is directly associated with the ability to operate and has also been proven to be associated with long-term survival [57]. The optimal chemotherapy regimen combined with targeted agents as neoadjuvant therapy for CRLM patients remains to be defined. We thus carry out a sub-group analysis stratified according to chemotherapy regimens. Our results find that irinotecan-based NCT plus targeted agents seems to improve hepatic resection rate and R0 hepatic resection rate when compared to NCT alone, while the addition of targeted agents to oxaliplatin-based NCT does not improve hepatic resection rate and R0 hepatic resection rate. A similar result have been observed in a large prospective clinical trials comparing FOLFOX versus FOLRIR as first-line treatment for metastatic colorectal cancer [58]. In that study of 220 unselected patients, FOLFIRI achieved a significantly higher rate of secondary surgery to remove metastases as compared to FOLFOX (22% vs. 9%; *P* = 0.02), with a higher R0 rate (13% vs 7%), which adding further validity to our findings. However, the results of our sub-group analysis regarding concurrent chemotherapy on efficacy of targeted agents are not solid since only one trial investigating FOLFIRI plus bevacizumab in resectable CRLM patients is included for analysis, thus more studies are still needed to assess the efficacy of irinotecan-based NCT plus targeted agents in those patients. We also investigate the efficacy of anti-EGFR-MoAbs in CRLM with K-ras wild-type. And the pooled ORR, hepatic resection and R0 hepatic resection rate for CRLM with K-ras wild-type is comparable to those for CRLM patients with or without K-ras wild-type. One possible explanation for this is that although these studies include CRLM patients with or without k-ras wild type, most of CRLM patients have k-ras wild type. For example, 81% CRLM patients had k-ras wild-type tumor in the trial conducted by Garufi C. et al [46]. Another possible explanation for this finding is that all RAS wild-type patients receiving anti-EGFR agents have a better efficacy than for patients with only KRAS exon 2 wild type, while patients in these previous trials did not detect other new RAS status including NRAS and exons 3 and 3 of KRAS, which might also be negative predictive biomarkers for anti-EGFR agents [57]. Moreover, we could not pool the results about quality of life (QoL) due to none of included trial reporting Qol results.

Several limitations exist in this analysis. First and most importantly, the application of formal meta-analytic methods to observational studies has been controversial [59]. One of the most important reasons for this is that the designs and populations of the studies are diverse, and that these differences may influence the pooled estimates. However, when no head-to-head comparison data available for NCT plus bevacizumab versus NCT alone, a meta-analysis of observational studies is one of the few methods for assessing efficacy [60]. Second, the study is a pooled analysis of primarily single arm prospective studies and retrospective series, with a small number of patients included that might have over-reported the benefit of preoperative treatments. The inclusion criteria also likely favor young, fit, and responder patients, a highly selected group of subjects with good prognostic indicators. Thirdly, this meta-analysis only considers published literature, and lack of individual patient data prevents us from adjusting the treatment effect according to disease and patient variables. Finally, we could not pool the results about QoL due to none of included trial reporting Qol results.

## MATERIALS AND METHODS

### Study design

We developed a protocol that defined inclusion criteria, search strategy, outcomes of interest, and analysis plan. The reporting of this systematic review adheres to the Preferred Reporting Items for Systematic Reviews and Meta-Analyses (PRISMA) statements [18].

### Identification and selection of studies

To identify studies for inclusion in our systematic review and meta-analysis, we did a broad search of four databases, including Embase, Medline, the Cochrane Central Register of Controlled Trials, and the Cochrane Database of Systematic Reviews, from the date of inception of every database to August 2014. The search included the following terms: “colorectal neoplasms”, “colorectal cancer”, “colorectal carcinoma”, “cetuximab”, “panitumumab”, “bevacizumab”, “aflibercept”, “targeted agents”, “neoadjuvant chemotherapy” and “perioperative chemotherapy”. Additional references were searched through manual searches of the reference lists and specialist journals. No language restrictions were applied.

To be eligible for inclusion in our systematic review and meta-analysis, study populations (referred to hereafter as cohorts) had to meet all the following criteria: 1) patients with colorectal liver metastasis; 2) treatment with neoadjuvant chemotherapy, NCT plus approved molecular agents (cetuximab, bevacizumab, panitumumab and aflibercept); 3) reported outcomes of interest (ie, objective response rate, overall resection rate, and R0 liver resection rate); and 4) from an original study (ie, randomized controlled trial, non-randomized clinical trial, observational studies, or case series).

### Data extraction

Two investigators screened the titles and abstracts of potentially relevant studies. We retrieved the full text of relevant studies for further review by the same two reviewers. A third senior investigator resolved any discrepancies between reviewers. If reviewers suspected an overlap of cohorts in a report, they contacted the corresponding author for clarification; we excluded studies with a clear overlap.

The same pair of reviewers extracted study details independently, using a standardized pilot-tested form. A third investigator reviewed all data entries. We extracted the following data: author, study design, study period, median age, interventions (neoadjuvant chemotherapy regimens and dose), sample size and outcomes of interest. We defined outcomes of interest as overall resection rate, R0 liver resection rate and objective response rate (ORR). ORR was defined as the sum of partial and complete response rates according to the Response Evaluation Criteria in Solid Tumors [19]. To assess quality, since we included non-comparative (uncontrolled) studies in our systematic review and meta-analysis, we used the Newcastle-Ottawa quality assessment scale [20]. We selected items that focused on representativeness of study patients, demonstration that the outcome of interest was not present at the start of the study, adequate assessment of outcome, sufficient length of follow-up to allow outcomes to arise, and adequacy of follow-up.

### Statistical analysis

We prespecified the analysis plan in the protocol. We analyzed all patients who started NCT or NCT plus targeted agents, regardless of their adherence to treatment. We calculated event rates of outcome (the proportion of patients who developed outcomes of interest) from the included cohorts for both NCT and NCT plus targeted agents. We pooled log-transformed event rates with DerSimonian and Laird random-effect models or using the Mantel-Haenszel test according to heterogeneity among included studies [21]. We used the test of interaction proposed by Altman and Bland to compare log-transformed rates of outcomes between NCT and NCT plus targeted agents [22]. A statistical test with a *p*-value less than 0.05 was considered significant. To account for the potential effect of publication bias, we used the Duval and Tweedie non-parametric trim-and-fill method [23]. To measure overall heterogeneity across the included cohorts, we calculated the *I*^ 2^ statistic, with *I*^ 2^ greater than 50% indicating high heterogeneity. We assessed potential publication bias by visual inspection of the symmetry of funnel plots and with the Egger regression asymmetry test. We did all statistical analyses with comprehensive meta-analysis software version 2.0(Biostat, Englewood, NJ, USA).

## CONCLUSIONS

Currently available clinical evidence indicates that NCT plus bevacizumab may be a feasible regimen for patients with CRLM in comparison with NCT alone. However, since the overall quantity and quality of data regarding NCT plus bevacizumab is poor and considering the risk of bias in comparisons between observation studies. The reported results do not allow for definite conclusions. As a result, prospective randomized studies, definitively comparing the survival and treatment toxicity between NCT plus bevacizumab and NCT alone, are strongly encouraged to clearly set the role of NCT plus bevacizumab in the treatment of CRLM patients.

## References

[1] Abdel-Rahman O, Fouad M (2014). Risk of mucocutaneous toxicities in patients with solid tumors treated with everolimus; a systematic review and meta-analysis. Expert Rev Anticancer Ther.

[2] Zhu AX, Kudo M, Assenat E, Cattan S, Kang YK, Lim HY, Poon RT, Blanc JF, Vogel A, Chen CL, Dorval E, Peck-Radosavljevic M, Santoro A, Daniele B, Furuse J, Jappe A (2014). Effect of everolimus on survival in advanced hepatocellular carcinoma after failure of sorafenib: the EVOLVE-1 randomized clinical trial. Jama.

[3] Wang W, Wang ZC, Shen H, Xie JJ, Lu H (2014). Dose-intensive versus dose-control chemotherapy for high-grade osteosarcoma: a meta-analysis. European review for medical and pharmacological sciences.

[4] Wesolowski R, Abdel-Rasoul M, Lustberg M, Paskell M, Shapiro CL, Macrae ER (2014). Treatment-related mortality with everolimus in cancer patients. Oncologist.

[5] Andre F, O'Regan R, Ozguroglu M, Toi M, Xu B, Jerusalem G, Masuda N, Wilks S, Arena F, Isaacs C, Yap YS, Papai Z, Lang I, Armstrong A, Lerzo G, White M (2014). Everolimus for women with trastuzumab-resistant, HER2-positive, advanced breast cancer (BOLERO-3): a randomised, double-blind, placebo-controlled phase 3 trial. Lancet Oncol.

[6] Aapro M, Andre F, Blackwell K, Calvo E, Jahanzeb M, Papazisis K, Porta C, Pritchard K, Ravaud A (2014). Adverse event management in patients with advanced cancer receiving oral everolimus: focus on breast cancer. Ann Oncol.

[7] Yamanaka K, Petrulionis M, Lin S, Gao C, Galli U, Richter S, Winkler S, Houben P, Schultze D, Hatano E, Schemmer P (2013). Therapeutic potential and adverse events of everolimus for treatment of hepatocellular carcinoma - systematic review and meta-analysis. Cancer Med.

[8] Beck JT, Hortobagyi GN, Campone M, Lebrun F, Deleu I, Rugo HS, Pistilli B, Masuda N, Hart L, Melichar B, Dakhil S, Geberth M, Nunzi M, Heng DY, Brechenmacher T, El-Hashimy M (2014). Everolimus plus exemestane as first-line therapy in HR(+), HER2(−) advanced breast cancer in BOLERO-2. Breast Cancer Res Treat.

[9] Pritchard KI, Burris HA, Ito Y, Rugo HS, Dakhil S, Hortobagyi GN, Campone M, Csoszi T, Baselga J, Puttawibul P, Piccart M, Heng D, Noguchi S, Srimuninnimit V, Bourgeois H, Gonzalez Martin A (2013). Safety and efficacy of everolimus with exemestane vs. exemestane alone in elderly patients with HER2-negative, hormone receptor-positive breast cancer in BOLERO-2. Clin Breast Cancer.

[10] Eberst L, Cropet C, Le Cesne A, Pautier P, Penel N, Adenis A, Chevreau C, Bay JO, Collard O, Cupissol D, Duffaud F, Gentet JC, Piperno-Neumann S, Marec-Berard P, Bompas E, Thyss A (2014). The off-label use of targeted therapies in sarcomas: the OUTC'S program. BMC Cancer.

[11] Lorenz M, Staib-Sebler E, Gog C, Proschek D, Jauch KW, Ridwelski K, Hohenberger W, Gassel HJ, Lehmann U, Vestweber KH, Padberg W, Zamzow K, Muller HH (2003). Prospective pilot study of neoadjuvant chemotherapy with 5-fluorouracil, folinic acid and oxaliplatin in resectable liver metastases of colorectal cancer. Analysis of 42 neoadjuvant chemotherapies. Zentralblatt fur Chirurgie.

[12] Robl B, Pauli C, Botter SM, Bode-Lesniewska B, Fuchs B (2015). Prognostic value of tumor suppressors in osteosarcoma before and after neoadjuvant chemotherapy. BMC Cancer.

[13] Liu Y, Ma YH, Sun ZZ, Rui YJ, Yin QD, Song S, Wei XM, Liu J, Liu XG, Hu KJ (2014). Effect of c-erbB2 overexpression on prognosis in osteosarcoma: evidence from eight studies. Tumour Biol.

[14] Jiang L, Tao C, He A (2013). Prognostic significance of p53 expression in malignant bone tumors: a meta-analysis. Tumour Biol.

[15] Jonker DJ, O'Callaghan CJ, Karapetis CS, Zalcberg JR, Tu D, Au HJ, Berry SR, Krahn M, Price T, Simes RJ, Tebbutt NC, van Hazel G, Wierzbicki R, Langer C, Moore MJ (2007). Cetuximab for the treatment of colorectal cancer. N Engl J Med.

[16] Haddox CL, Han G, Anijar L, Binitie O, Letson GD, Bui MM, Reed DR (2014). Osteosarcoma in pediatric patients and young adults: a single institution retrospective review of presentation, therapy, and outcome. Sarcoma.

[17] Khoury JF, Ben-Arush MW, Weintraub M, Waldman E, Futerman B, Vlodavsky E, Postovsky S (2014). Alkaline phosphatase level change in patients with osteosarcoma: its role as a predictive factor of tumor necrosis and clinical outcome. The Israel Medical Association journal : IMAJ.

[18] Ferrari S, Ruggieri P, Cefalo G, Tamburini A, Capanna R, Fagioli F, Comandone A, Bertulli R, Bisogno G, Palmerini E, Alberghini M, Parafioriti A, Linari A, Picci P, Bacci G (2012). Neoadjuvant chemotherapy with methotrexate, cisplatin, and doxorubicin with or without ifosfamide in nonmetastatic osteosarcoma of the extremity: an Italian sarcoma group trial ISG/OS-1. J Clin Oncol.

[19] Vauthey JN, Pawlik TM, Ribero D, Wu TT, Zorzi D, Hoff PM, Xiong HQ, Eng C, Lauwers GY, Mino-Kenudson M, Risio M, Muratore A, Capussotti L, Curley SA, Abdalla EK (2006). Chemotherapy regimen predicts steatohepatitis and an increase in 90-day mortality after surgery for hepatic colorectal metastases. J Clin Oncol.

[20] Womer RB, West DC, Krailo MD, Dickman PS, Pawel BR, Grier HE, Marcus K, Sailer S, Healey JH, Dormans JP, Weiss AR (2012). Randomized controlled trial of interval-compressed chemotherapy for the treatment of localized Ewing sarcoma: a report from the Children's Oncology Group. J Clin Oncol.

[21] Bernthal NM, Federman N, Eilber FR, Nelson SD, Eckardt JJ, Eilber FC, Tap WD (2012). Long-term results (>25 years) of a randomized, prospective clinical trial evaluating chemotherapy in patients with high-grade, operable osteosarcoma. Cancer.

[22] Ebb D, Meyers P, Grier H, Bernstein M, Gorlick R, Lipshultz SE, Krailo M, Devidas M, Barkauskas DA, Siegal GP, Ferguson WS, Letson GD, Marcus K, Goorin A, Beardsley P, Marina N (2012). Phase II trial of trastuzumab in combination with cytotoxic chemotherapy for treatment of metastatic osteosarcoma with human epidermal growth factor receptor 2 overexpression: a report from the children's oncology group. J Clin Oncol.

[23] Duval S, Tweedie R (2000). Trim and fill: A simple funnel-plot-based method of testing and adjusting for publication bias in meta-analysis. Biometrics.

[24] Piccart M, Hortobagyi GN, Campone M, Pritchard KI, Lebrun F, Ito Y, Noguchi S, Perez A, Rugo HS, Deleu I, Burris HA, Provencher L, Neven P, Gnant M, Shtivelband M, Wu C (2014). Everolimus plus exemestane for hormone-receptor-positive, human epidermal growth factor receptor-2-negative advanced breast cancer: overall survival results from BOLERO-2dagger. Ann Oncol.

[25] Zhong A, Xiong X, Shi M, Xu H (2015). The efficacy and safety of pemetrexed-based doublet therapy compared to pemetrexed alone for the second-line treatment of advanced non-small-cell lung cancer: an updated meta-analysis. Drug design, development and therapy.

[26] Suenaga M, Fujimoto Y, Matsusaka S, Shinozaki E, Akiyoshi T, Nagayama S, Fukunaga Y, Oya M, Ueno M, Mizunuma N, Yamaguchi T (2015). Perioperative FOLFOX4 plus bevacizumab for initially unresectable advanced colorectal cancer (NAVIGATE-CRC-01). Onco Targets Ther.

[27] Parks R, Gonen M, Kemeny N, Jarnagin W, D'Angelica M, DeMatteo R, Garden OJ, Blumgart LH, Fong Y (2007). Adjuvant chemotherapy improves survival after resection of hepatic colorectal metastases: analysis of data from two continents. Journal of the American College of Surgeons.

[28] Pietrantonio F, Mazzaferro V, Miceli R, Cotsoglou C, Melotti F, Fanetti G, Perrone F, Biondani P, Muscara C, Di Bartolomeo M, Coppa J, Maggi C, Milione M, Tamborini E, de Braud F (2015). Pathological response after neoadjuvant bevacizumab- or cetuximab-based chemotherapy in resected colorectal cancer liver metastases. Med Oncol.

[29] Primrose J, Falk S, Finch-Jones M, Valle J, O'Reilly D, Siriwardena A, Hornbuckle J, Peterson M, Rees M, Iveson T, Hickish T, Butler R, Stanton L, Dixon E, Little L, Bowers M (2014). Systemic chemotherapy with or without cetuximab in patients with resectable colorectal liver metastasis: the New EPOC randomised controlled trial. Lancet Oncol.

[30] Ravaud A, Barrios CH, Alekseev B, Tay MH, Agarwala SS, Yalcin S, Lin CC, Roman L, Shkolnik M, Anak O, Gogov S, Pelov D, Louveau AL, Melichar B (2015). RECORD-2: phase II randomized study of everolimus and bevacizumab versus interferon alpha-2a and bevacizumab as first-line therapy in patients with metastatic renal cell carcinoma. Ann Oncol.

[31] Masi G, Loupakis F, Salvatore L, Fornaro L, Cremolini C, Cupini S, Ciarlo A, Del Monte F, Cortesi E, Amoroso D, Granetto C, Fontanini G, Sensi E, Lupi C, Andreuccetti M, Falcone A (2010). Bevacizumab with FOLFOXIRI (irinotecan, oxaliplatin, fluorouracil, and folinate) as first-line treatment for metastatic colorectal cancer: a phase 2 trial. Lancet Oncol.

[32] Folprecht G, Gruenberger T, Bechstein WO, Raab HR, Lordick F, Hartmann JT, Lang H, Frilling A, Stoehlmacher J, Weitz J, Konopke R, Stroszczynski C, Liersch T, Ockert D, Herrmann T, Goekkurt E (2010). Tumour response and secondary resectability of colorectal liver metastases following neoadjuvant chemotherapy with cetuximab: the CELIM randomised phase 2 trial. Lancet Oncol.

[33] Ye LC, Liu TS, Ren L, Wei Y, Zhu DX, Zai SY, Ye QH, Yu Y, Xu B, Qin XY, Xu J (2013). Randomized controlled trial of cetuximab plus chemotherapy for patients with KRAS wild-type unresectable colorectal liver-limited metastases. J Clin Oncol.

[34] Gruenberger B, Tamandl D, Schueller J, Scheithauer W, Zielinski C, Herbst F, Gruenberger T (2008). Bevacizumab, capecitabine, and oxaliplatin as neoadjuvant therapy for patients with potentially curable metastatic colorectal cancer. J Clin Oncol.

[35] Wang X, Ji A, Zhu Y, Liang Z, Wu J, Li S, Meng S, Zheng X, Xie L (2015). A meta-analysis including dose-response relationship between night shift work and the risk of colorectal cancer. Oncotarget.

[36] Shameem R, Lacouture M, Wu S (2015). Incidence and risk of high-grade stomatitis with mTOR inhibitors in cancer patients. Cancer Invest.

[37] Shameem R, Hamid MS, Wu S (2015). Risk of anemia attributable to everolimus in patients with cancer: a meta-analysis of randomized controlled trials. Anticancer Res.

[38] Chaudhury P, Hassanain M, Bouganim N, Salman A, Kavan P, Metrakos P (2010). Perioperative chemotherapy with bevacizumab and liver resection for colorectal cancer liver metastasis. HPB (Oxford).

[39] Iacovelli R, Pietrantonio F, Farcomeni A, Maggi C, Palazzo A, Ricchini F, de Braud F, Di Bartolomeo M (2014). Chemotherapy or targeted therapy as second-line treatment of advanced gastric cancer. A systematic review and meta-analysis of published studies. PLoS One.

[40] Malik H, Khan AZ, Berry DP, Cameron IC, Pope I, Sherlock D, Helmy S, Byrne B, Thompson M, Pulfer A, Davidson B (2015). Liver resection rate following downsizing chemotherapy with cetuximab in metastatic colorectal cancer: UK retrospective observational study. Eur J Surg Oncol.

[41] Vera R, Gomez Dorronsoro M, Lopez-Ben S, Viudez A, Queralt B, Hernandez I, Ortiz-Duran MR, Zazpe C, Soriano J, Amat I, Herrera Cabezon J, Diaz E, Codina-Barreras A, Hernandez-Yague X, Quera A, Figueras J (2014). Retrospective analysis of pathological response in colorectal cancer liver metastases following treatment with bevacizumab. Clin Transl Oncol.

[42] Constantinidou A, Cunningham D, Shurmahi F, Asghar U, Barbachano Y, Khan A, Mudan S, Rao S, Chau I (2013). Perioperative chemotherapy with or without bevacizumab in patients with metastatic colorectal cancer undergoing liver resection. Clin Colorectal Cancer.

[43] Ji JH, Park SH, Lee J, Kim TW, Hong YS, Kim KP, Kim SY, Baek JY, Kang HJ, Shin SJ, Shim BY, Park YS (2013). Prospective phase II study of neoadjuvant FOLFOX6 plus cetuximab in patients with colorectal cancer and unresectable liver-only metastasis. Cancer Chemother Pharmacol.

[44] Nordlinger B, Guiguet M, Vaillant JC, Balladur P, Boudjema K, Bachellier P, Jaeck D (1996). Surgical resection of colorectal carcinoma metastases to the liver. A prognostic scoring system to improve case selection, based on 1568 patients. Association Francaise de Chirurgie. Cancer.

[45] Nasti G, Piccirillo MC, Izzo F, Ottaiano A, Albino V, Delrio P, Romano C, Giordano P, Lastoria S, Caraco C, de Lutio di Castelguidone E, Palaia R, Daniele G, Aloj L, Romano G, Iaffaioli RV (2013). Neoadjuvant FOLFIRI+bevacizumab in patients with resectable liver metastases from colorectal cancer: a phase 2 trial. Br J Cancer.

[46] Garufi C, Torsello A, Tumolo S, Ettorre GM, Zeuli M, Campanella C, Vennarecci G, Mottolese M, Sperduti I, Cognetti F (2010). Cetuximab plus chronomodulated irinotecan, 5-fluorouracil, leucovorin and oxaliplatin as neoadjuvant chemotherapy in colorectal liver metastases: POCHER trial. Br J Cancer.

[47] Bertolini F, Malavasi N, Scarabelli L, Fiocchi F, Bagni B, Del Giovane C, Colucci G, Gerunda GE, Depenni R, Zironi S, Fontana A, Pettorelli E, Luppi G, Conte PF (2011). FOLFOX6 and bevacizumab in non-optimally resectable liver metastases from colorectal cancer. Br J Cancer.

[48] Nordlinger B, Sorbye H, Glimelius B, Poston GJ, Schlag PM, Rougier P, Bechstein WO, Primrose JN, Walpole ET, Finch-Jones M, Jaeck D, Mirza D, Parks RW, Collette L, Praet M, Bethe U (2008). Perioperative chemotherapy with FOLFOX4 and surgery versus surgery alone for resectable liver metastases from colorectal cancer (EORTC Intergroup trial 40983): a randomised controlled trial. Lancet.

[49] Fong Y, Fortner J, Sun RL, Brennan MF, Blumgart LH (1999). Clinical score for predicting recurrence after hepatic resection for metastatic colorectal cancer: analysis of 1001 consecutive cases. Ann Surg.

[50] Rees M, Tekkis PP, Welsh FK, O'Rourke T, John TG (2008). Evaluation of long-term survival after hepatic resection for metastatic colorectal cancer: a multifactorial model of 929 patients. Ann Surg.

[51] Beppu T, Emi Y, Tokunaga S, Oki E, Shirabe K, Ueno S, Kuramoto M, Kabashima A, Takahashi I, Samura H, Eguchi S, Akagi Y, Natsugoe S, Ogata Y, Kakeji Y, Baba H (2014). Liver resectability of advanced liver-limited colorectal liver metastases following mFOLFOX6 with bevacizumab (KSCC0802 Study). Anticancer Res.

[52] Uetake H, Yasuno M, Ishiguro M, Kameoka S, Shimada Y, Takahashi K, Watanabe T, Muro K, Baba H, Yamamoto J, Mizunuma N, Tamagawa H, Mochizuki I, Kinugasa Y, Kikuchi T, Sugihara K (2015). A multicenter phase II trial of mFOLFOX6 plus bevacizumab to treat liver-only metastases of colorectal cancer that are unsuitable for upfront resection (TRICC0808). Annals of surgical oncology.

[53] Wong R, Cunningham D, Barbachano Y, Saffery C, Valle J, Hickish T, Mudan S, Brown G, Khan A, Wotherspoon A, Strimpakos AS, Thomas J, Compton S, Chua YJ, Chau I (2011). A multicentre study of capecitabine, oxaliplatin plus bevacizumab as perioperative treatment of patients with poor-risk colorectal liver-only metastases not selected for upfront resection. Ann Oncol.

[54] Gruenberger T, Bridgewater J, Chau I, Garcia Alfonso P, Rivoire M, Mudan S, Lasserre S, Hermann F, Waterkamp D, Adam R (2015). Bevacizumab plus mFOLFOX-6 or FOLFOXIRI in patients with initially unresectable liver metastases from colorectal cancer: the OLIVIA multinational randomised phase II trial. Ann Oncol.

[55] Klinger M, Tamandl D, Eipeldauer S, Hacker S, Herberger B, Kaczirek K, Dorfmeister M, Gruenberger B, Gruenberger T (2010). Bevacizumab improves pathological response of colorectal cancer liver metastases treated with XELOX/FOLFOX. Annals of surgical oncology.

[56] Ribero D, Wang H, Donadon M, Zorzi D, Thomas MB, Eng C, Chang DZ, Curley SA, Abdalla EK, Ellis LM, Vauthey JN (2007). Bevacizumab improves pathologic response and protects against hepatic injury in patients treated with oxaliplatin-based chemotherapy for colorectal liver metastases. Cancer.

[57] Feng QY, Wei Y, Chen JW, Chang WJ, Ye LC, Zhu DX, Xu JM (2014). Anti-EGFR and anti-VEGF agents: important targeted therapies of colorectal liver metastases. World J Gastroenterol.

[58] Tournigand C, Andre T, Achille E, Lledo G, Flesh M, Mery-Mignard D, Quinaux E, Couteau C, Buyse M, Ganem G, Landi B, Colin P, Louvet C, de Gramont A (2004). FOLFIRI followed by FOLFOX6 or the reverse sequence in advanced colorectal cancer: a randomized GERCOR study. J Clin Oncol.

[59] Iwamoto Y, Tanaka K, Isu K, Kawai A, Tatezaki S, Ishii T, Kushida K, Beppu Y, Usui M, Tateishi A, Furuse K, Minamizaki T, Kawaguchi N, Yamawaki S (2009). Multiinstitutional phase II study of neoadjuvant chemotherapy for osteosarcoma (NECO study) in Japan: NECO-93J and NECO-95J. Journal of orthopaedic science: official journal of the Japanese Orthopaedic Association.

[60] Granowetter L, Womer R, Devidas M, Krailo M, Wang C, Bernstein M, Marina N, Leavey P, Gebhardt M, Healey J, Shamberger RC, Goorin A, Miser J, Meyer J, Arndt CA, Sailer S (2009). Dose-intensified compared with standard chemotherapy for nonmetastatic Ewing sarcoma family of tumors: a Children's Oncology Group Study. J Clin Oncol.

